# Present Scenario of Bioconjugates in Cancer Therapy: A Review

**DOI:** 10.3390/ijms20215243

**Published:** 2019-10-23

**Authors:** Aishani Wadhawan, Mary Chatterjee, Gurpal Singh

**Affiliations:** 1 Biotechnology Branch, University Institute of Engineering and Technology, Sector-25, South Campus, Panjab University, Chandigarh Pin code-160014, India; aishani74@gmail.com; 2 Department of Pharmaceutical Sciences, University Institute of Pharmaceutical Sciences, Sector-14, Panjab University, Chandigarh Pin code-160014, India; gurpal79@pu.ac.in

**Keywords:** bioconjugate, cancer therapy, drug delivery, nanoparticles, nanomedicine, therapeutics, imaging

## Abstract

Cancer is one of the deadliest diseases and poses a risk to people all over the world. Surgery, chemo, and radiation therapy have been the only options available until today to combat this major problem. Chemotherapeutic drugs have been used for treatment for more than 50 years. Unfortunately, these drugs have inherent cytotoxicities and tumor cells have started inducing resistance against these drugs. Other common techniques such as surgery and radiotherapy have their own drawbacks. Therefore, such techniques are incompetent tools to alleviate the disease efficiently without any adverse effects. This scenario has inspired researchers to develop alternative techniques with enhanced therapeutic effects and minimal side effects. Such techniques include targeted therapy, liposomal therapy, hormonal therapy, and immunotherapy, etc. However, these therapies are expensive and not effective enough. Furthermore, researchers have conjugated therapeutic agents or drugs with different molecules, delivery vectors, and/or imaging modalities to combat such problems and enhance the therapeutic effect. This conjugation technique has led to the development of bioconjugation therapy, in which at least one molecule is of biological origin. These bioconjugates are the new therapeutic strategies, having prospective synergistic antitumor effects and have potency to overcome the complications being produced by chemo drugs. Herein, we provide an overview of various bioconjugates developed so far, as well as their classification, characteristics, and targeting approach for cancer. Additionally, the most popular nanostructures based on their organic or inorganic origin (metallic, magnetic, polymeric nanoparticles, dendrimers, and silica nanoparticles) characterized as nanocarriers are also discussed. Moreover, we hope that this review will provide inspiration for researchers to develop better bioconjugates as therapeutic agents.

## 1. Introduction

Cancer is a burgeoning problem related to public health and a global threat to the human race. According to the Globocan2018 study, 18.1 million new cancer cases and 9.6 million cancer deaths were reported in 2018 [1]. Cancer-related deaths increased by about 17% in comparison to data available in 2012 [2]. Irrespective of sex, people are mostly diagnosed with lung cancer, which is also the major cause of cancer death among males. However, breast cancer is most prevalent in females and is also life threatening [2].

**Figure 1:**
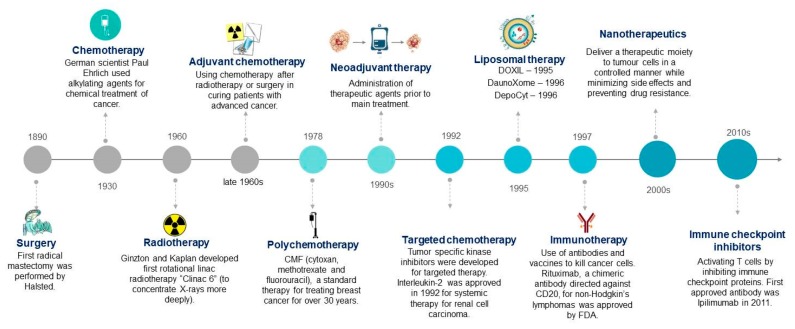
Evolution of cancer therapy techniques to date [3,4]. (Cliparts are adapted and modified from clipartlibrary.com).

All types of cancer therapies which have evolved to date are depicted in Figure 1. The most common and conventional therapies against this deadly disease include surgery, radiation, and chemotherapy. These conventional therapeutics have several side effects, which causes a lot of physical as well as psychological stress among patients [5]. Chemotherapeutic drugs induce certain toxicity in our body, including hematotoxicity, cardiotoxicity, gastrointestinal toxicity, neurotoxicity, nephrotoxicity, and hair follicle toxicity, etc. [6]. These drugs target rapidly multiplying cells, which leads to inefficiency in differentiating between cancerous and normal cells [7]. This restricts the maximum allowable dose of drugs. On the other hand, these chemotherapeutic drugs get rapidly eliminated from the body through renal or other metabolic processes. Therefore, administration of a high dosage of drug is required to avoid rapid elimination and for widespread distribution of the drug to the targeted area, which is not economical and causes undesirable toxicity [8].

Subsequent advances and development have led to various alternative therapies to overcome such problems. These include liposomal therapy, targeted therapy, immunotherapy, hormone therapy, and stem cell therapy. In liposomal therapy, drugs are encapsulated inside vesicles made up of a phospholipid bilayer, known as liposomes. This therapy emerged in the 1990s and minimizes some of the side effects of chemotherapy. The first drug-encapsulated liposome approved by the FDA was DaunoXome (daunorubicin liposomal) in 1996, after which came Doxil (doxorubicin liposomal) (2004) and DepoCyt (cytarabine liposomal), etc. Simultaneously, with the discovery of developing antibodies or other targeted molecules artificially, inhibitor and targeted therapy emerged. Rituximab (Rituxan^TM^) (1997) was the first approved monoclonal chimeric antibody which was targeted against CD20 of non-Hodgkin’s lymphomas and Sorafenib (2005) was the first FDA-approved kinase inhibitor drug which was developed for the treatment of renal cell cancer (RCC) [3]. However, one of the main disadvantages of targeted therapy is that antibodies are proteins which undergo enzymatic digestion inside the body and are converted into lower molecular weight fragments. These fragments are usually cleared through the renal system. Therefore, conjugation of proteins with nanocarriers such as polymer enhances their solubility, stability, and immunological profile [9].

**Figure 2:**
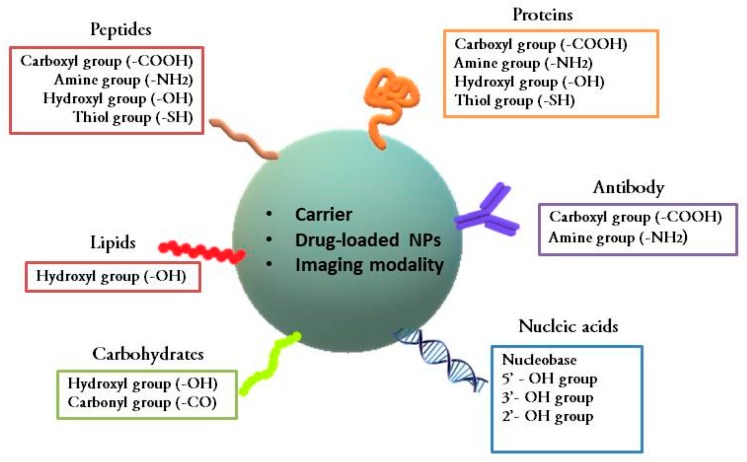
Chemical linkages in bioconjugates. Nanoparticles are abbreviated as NPs.

Furthermore, with the advancement in nanostructure-based therapeutics and diagnostic agents, certain conjugated nanoformulations and nanoparticles have been developed for treating cancer. Some examples are Genexol-PM (paclitaxel loaded in a polyethylene glycol-polylactic acid copolymer (PEG-PLA)), Zinostatin Stimalmer (polymer protein conjugate), Abraxane (paclitaxel conjugated with albumin), NanoTherm nanoparticles (aminosilane-coated iron oxide), Gliadel (biodegradable polymeric wafer loaded with carmustine), and Feridex/Endorem (superparamagnetic iron oxide nanoparticles (SPIONs) coated with dextrane), etc. [3]. Drug dosages used in developing these nanomedicines are lower than individual chemotherapy. This reduces the side effects related to large doses of drugs and enhances their therapeutic effect and safety profiles. These nano-delivery systems contain drugs, therapeutic agents, and imaging agents which are either conjugated, encapsulated, dispersed, or adsorbed [10]. These nanoformulations have a high surface area to volume ratio, good stability, and enhanced permeability and retention (EPR) [11]. This technique is a traditional method for delivering drugs via nanocarriers in which the drug associated with the carrier is aggregated into the cancer tissues. The structural abnormality in blood vessels near cancer cells leads to increased tissue permeability, delivery, and retention of drug molecules inside the cancer cells. This passive delivery of drugs is not very efficient and eventually, the drug is released back towards the high concentration area into the blood [10]. Hence, the bioconjugation technique can be used to deliver drugs selectively to the targeted cancer site. Bioconjugation consists of linking two molecules, usually via a covalent bond. Here, at least one molecule should be of biological origin or a biomolecule [12]. It is a tool that bridges chemistry and biology [13]. In the case of cancer therapeutic agents, these biologically originated molecules used for conjugation are primarily the ligands that target tumor-specific antigens [14,15]. Alternatively, they can be peptides [16,17], glycoproteins [18], aptamers [19,20], or interferons [21], etc.; these all have anticancerous properties. The unique advantage of bioconjugates is their ability to selectively deliver therapeutics to pathological sites and to increase the retention of the molecule in the blood circulation system. Their delivery mechanism is based on active delivery of drugs [22]. The chemical functional groups utilized for the linking of various bioconjugates have been described in Figure 2. These bioconjugates are one of the most fascinating thrust areas undergoing various in vitro and in vivo studies. Examples of some of the bioconjugates developed so far have been given in Table 1.

Different types of biomolecules conjugated in cioconjugates are:

1. Ligand receptors (targeted anticancers)—antibodies, aptamers, and peptides
2. Anticancer agents—peptides, glycoproteins, interferons, and biosurfactants

In targeted anticancer therapy, ligands are conjugated to the moieties that carry the chemotherapeutic agent [10]. Based on this ligand–receptor interaction, different combinations of bioconjugates have been developed. These receptors are cell surface specific markers which are overexpressed on cancerous cells [43]. This method utilizes the advantage of the high affinity of ligands to overexpressed cancer cell receptors or cancer-specific antigens in cancer cells [10]. Cell surface markers like human epidermal growth factor receptor (c-erbB-2 or HER-2) [22] or luteinizing releasing hormone (LHRH) receptors [36] have been reported to be overexpressed in cancer cells. This therapy is based on the selective delivery of an effective anticancer agent. Another type of ligand used is the peptide ligand, which comprises amazing properties. The structure of peptide ligands is simple, having less immunogenicity, and has excellent tissue permeability. They can be synthesized at a large scale by chemical synthesis. Their production is cost effective in comparison to high-cost monoclonal antibodies [44]. However, these peptides are less stable in vivo and are prone to proteolytic digestion. This limitation has been overcome by substituting one or more amino acids with unnatural D-amino acids. Gonadotropin-releasing hormone (GnRH) antagonist is one example of a modified peptide. Schuster et al. have used a derivative of GnRH, known as GNRH-III, conjugated with daunorubicin, a chemotherapeutic drug. GnRH receptors are reported to be overexpressed in ovarian, breast, colorectal, prostate, and endometrial cancer [44].

Biomolecules having therapeutic effects (interferons, peptides, and glycoproteins) are also being used in bioconjugates. For instance, Montganer et al. have studied the potency of interferons as a therapeutic agent by conjugating them with hyaluronic acid (HA). They used HA as a carrier as well as a targeted ligand that selectively interacted with CD44-overexpressed cancer cells [21]. Other important molecules used for targeted cancer therapy are glycoproteins and agglutinins, which bind specifically to sugars. Agglutinins, a class of glycoproteins, can bind to receptors present on cancer cell membranes and cause agglutination or aggregation in these cells, which generates cytotoxicity and leads to apoptosis through caspase activation [18]. Nucleic acid (aptamer, miRNA, and siRNA) bioconjugates are also extensively used in cancer therapy. They act as a targeted ligand as well as a therapeutic agent. They suppress cancer proliferation by downregulating the respective oncoproteins and lead to apoptosis [19].

**Figure 3:**
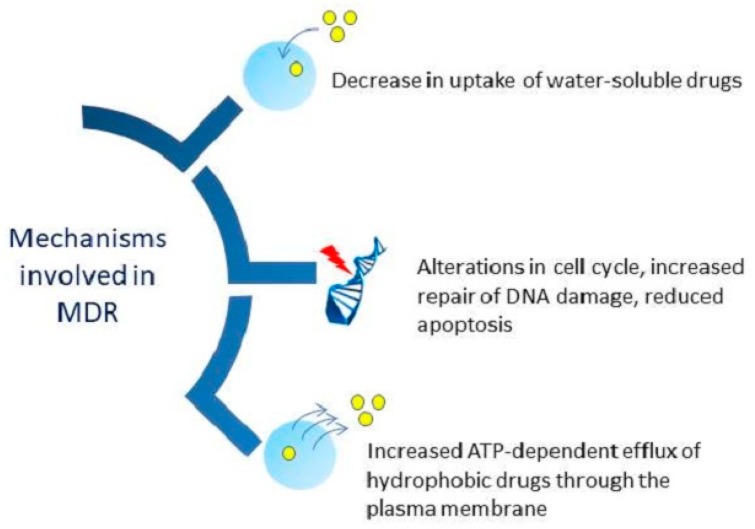
Different mechanisms involved in multi-drug resistance (MDR) by tumor cells [48].

However, another major problem faced currently in cancer therapy is drug resistance caused by genetic alterations in tumor and epigenetic changes [45]. Multi-drug resistant (MDR) cancer cells cannot be cured by normal standard cytotoxic agents [46,47]. In drug-resistant cancer cells, overexpression of cell-membrane transporters increases the efflux of the cytotoxic drugs from cells and thereby lowers the drug retention in cancer cells [46]. Probable mechanisms of drug resistance have has been explained in Figure 3.

Researchers have synthesized bioconjugates to overcome MDR by conjugating certain molecules, such as siRNA, which inhibit drug efflux pumps. Meng et al. conjugated a doxorubicin-encapsulated drug delivery system (mesoporous silica nanoparticles) with Pgp siRNA that knocked down the Pgp gene expression. This system restored the drug concentration in the cancerous cells, which is essential to induce apoptosis [28]. Qi et al. conjugated siRNA that targets ABCG2 (ATP-binding cassette, subfamily G, member 2, MDR tumor) with mesoporous silica nanoparticles encapsulated with drugs [49]. They compared the effects of bioconjugated nanoparticles with individual drugs on CD133+ tumors in mice. The authors showed that bioconjugated drug-loaded nanoparticles are more effective against tumors than individual drugs. This experiment was observed for three chemotherapeutic drugs, namely, cisplatin, paclitaxel, and 5-fluorouracil [49]. Recently, quantum dots (QDs) functionalized with a cell-penetrating peptide (JB434) have been used to tackle drug resistance in H69AR cells. JB434 is an arginine-rich peptide which is highly positively charged. It is attracted to the negatively charged plasma membrane. This helps in the internalization of QDs via the endocytic pathway. Here, in this study, doxorubicin was attached on the surface of QDs via a novel peptide through an ester linkage. This peptide-drug conjugate helps in enzymatic cleavage by esterases found in the endocytic pathway. The bioconjugated system was found to be more effective against drug-resistant cell lines when compared to the drug alone [41,50].

Another type of immunotherapy has emerged in the 2010s which uses immune checkpoint inhibitors to treat cancer (Figure 1). Our immune system uses a powerful weapon, cytotoxic T-cells, to fight against cancer. Immune checkpoint receptors present on immune cell surfaces help to control immune responses against normal host cells and keep T-cells inactive until they are needed. Cancer cells also possess ligands to these checkpoint receptors like normal cells. This keeps immune cells in their inactive form. Immune checkpoint inhibitors are drugs or monoclonal antibodies that block T-cell checkpoint antigens and freeze them in their active form to kill cancer cells. Cytotoxic T lymphocyte-associated antigen 4 (CTLA-4) and the programmed cell death protein 1 pathway (PD-1/PD-L1) are checkpoints which have been targeted to block by antibodies [51,52]. Ipilimumab was the first monoclonal antibody globally approved to target against CTLA-4 [4]. Other antibodies which have entered clinical trials are tremelimumab (against CTLA-4), nivolumab, and pembrolizumab (against PD-1), etc. [51]. However, clinical studies have reported adverse effects, such as diarrhea, fatigue, pruritus, and colitis, etc. in patients treated with ipilimumab, nivolumab, and their combination [53]. Conjugation of immune checkpoint inhibitor antibodies (Abs) with certain molecules that can enhance the retention of these Abs in cancer cells and reduce their exposure to other normal cells is desirable [54]. Ishihara et al. conjugated immune checkpoint blockade antibodies (PD-L1 and CTLA-4) with a peptide which had an affinity for the extracellular matrix (ECM). This peptide was derived from placenta growth factor-2. They examined the adverse events of conjugated Abs in a B16F10 mice model and observed less systemic toxicity when compared to unmodified Abs. Also, antitumor efficiency of these modified antibodies was also increased compared to the unmodified one [54]. In 2019, they conjugated a collagen binding domain (CBD) to immune checkpoint inhibitors and cytokines (interleukin-2) to further enhance the efficacy of the system and improve upon the adverse effects. Due to leaky vasculature of tumors, collagen is exposed to molecules in the blood stream. Hence, this system was able to target tumors via blood routes with enhanced anti-tumor efficacy and reduced cytotoxicity and adverse events [55]. 

These bioconjugates have the potential to revolutionize cancer therapy and diagnosis techniques; however, further research and clinical trials are needed to make them commercial. Cancer therapy should be effective, with less adverse effects, and it should be affordable. Bioconjugation may be near to a perfect technique for cancer treatment and one able to conquer this deadly disease.

## 2. Nanomaterials as Carriers of Biomolecules in Conjugates

**Figure 4:**
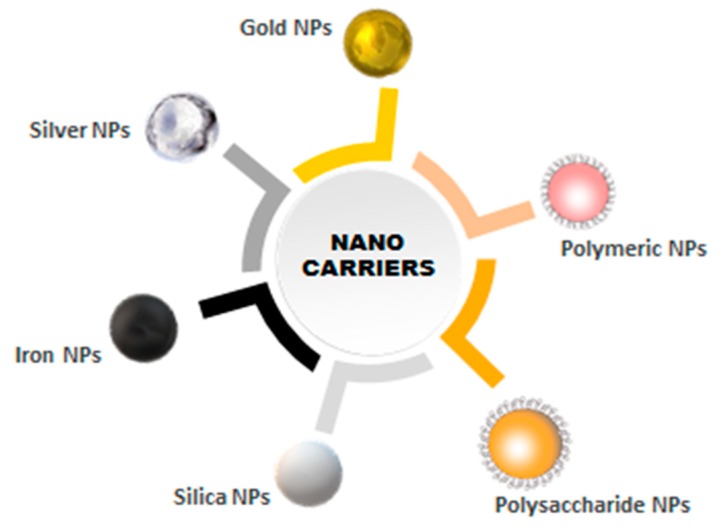
Different types of nanocarriers used for the preparation of bioconjugates.

Researchers have used various inorganic and organic nanocarriers for bioconjugation [38]. Such nanocarriers include nanoparticles of different metals, carbon nanotubes, quantum dots, dendrimers, hydrogels, and other nanocarriers of biological origin. Some of these nanocarriers have been depicted in Figure 4. The multifunctional properties of these nanocarriers could be employed for targeting, tracking, and therapy [11]. A good number of these products are under preclinical and clinical trials [38].

### 2.1. Inorganic Nanocarriers

#### 2.1.1. Metallic Nanoparticles

##### Gold Nanoparticles (GNPs)

GNPs are nanocarriers commonly used for biological purposes because these NPs are optically stable, easy to use, less cytotoxic, and their surface can be easily functionalized [56,57]. Gold nano-sized particles can be synthesized in different shapes and have distinct physicochemical properties. They can be used as contrast agents for diagnostic purposes, as photothermal agents, and as radio-sensitizers for therapy purposes [26]. Gold nanoparticles can be used as nanoparticles [22,26,57], nanospheres [27,56], nanorods [56,58,59], nanoshells [60], nanoclusters [61], and nanocages [62]. They show near-infrared (NIR) resonance due to their exceptional morphology. Their optical properties are very useful in the biomedical field. NIR light does not hinder nearby tissues and other components such as water and hemoglobin. Hence, GNPs can be a better choice as a less invasive imaging and treatment modality. Moreover, during the treatment process lasers specifically excite the molecules at the focal plane. Through this, high resolution images are produced [22,63]. GNPs can be conjugated with certain proteins or molecules for targeted therapy either through covalent attachment or non-covalent interactions (hydrophobic or electrostatic interactions) or can be specifically bound via a cofactor [22,64].

Antibodies are immobilized on GNPs by the F_c_ region and their antigen recognition sites (F_ab_ regions) are easily available to the targeted antigens [22]. Sun et al. developed immunoconjugated gold nanoparticles using protein G as a cofactor which can immobilize various IgG antibodies, thus generating a versatile targeted therapy system. These nanoparticles are considered a potential tool for photothermal cancer therapy due to their unique NIR absorption [22]. Researchers also use proteins like albumin for cancer targeting and therapy. Albumin accumulates in malignant tissue sites which have leaky capillaries and are rapidly taken up and metabolized by nutrient-starved cancer cells. For instance, Mocan et al. used albumin (BSA) to target liver cancer cells by conjugating it to gold nanoparticles. Significant necrosis was observed on the tumor cells after laser therapy, which had not affected the nearby parenchyma cells [65]. In a study by Liang et al., hollow gold nanospheres were bioconjugated with an anti-c-Met antibody to target cervical cancer cells. They suggested using this conjugate in combination with X-ray radiation therapy for the treatment of cervical cancer to reduce the dose of radiations. According to the authors, these hollow gold nanospheres have strong therapeutic potential as they have a high zeta coefficient, are easy to synthesize, and can be easily modified. MET proto-oncogene receptor tyrosine kinase (c‑Met) is the receptor for hepatocyte growth factor (HGF) and is overexpressed in cancer cells [27].

Researchers have also used various biomolecules or biopolymers to stabilize GNPs and increase their biocompatibility. Chitosan, alginate, gelatin, dextran, copolymers, and biosurfactants, etc., are examples of such molecules used as stabilizing agents for gold nanoparticles [66]. Recently, Spadavecchia et al. used PEG as a stabilizing agent for GNPs, loaded them with doxorubicin, and decorated them with a polyclonal antibody to target pancreatic cancer cells [67]. Further authors bioconjugated these PEGylated AuNPs with lactose-modified chitosan (CTL) and investigated their interaction with Galectin 1 protein, which is highly expressed in certain tumors such as colon, breast, lung, head and neck, ovarian, prostate cancer, and Hodgkin’s lymphoma. They concluded that there is a high binding affinity between Gal 1 protein and CTL-PEGylated GNPs which can be further utilized for cancer treatment [68].

##### Silver Nanoparticles (AgNPs)

AgNPs have been used for cancer therapy and imaging purposes [18]. They have unique physico-chemical properties such as high thermal conductivity, chemical stability, plasmonic properties, and antibacterial ability [69,70,71]. They possess antifungal, anti-inflammatory, antiviral, and antiplatelet activity [70]. AgNPs are potential tools as anticancer agents for diagnostic purposes because their surface can be easily functionalized, they have a high surface to volume ratio, and they have amazing optical properties [70]. These particles enter inside the cell, damage mitochondria, and reduce the cellular ATP content. They also generate oxidative stress, inducing cytochrome c-mediated apoptosis, and increase the production of reactive oxygen species (ROS). All these events arrest the cell cycle in the G-2/M phase and damage DNA [72]. However, in order to increase the site specificity of these AgNPs for targeting cancer cells, they should be conjugated with targeting systems [18]. Pimentel et al. synthesized AgNPs bioconjugated with soybean agglutinin (SBA) for treatment of breast cancer cells and compared the in vitro cytotoxicity effect of free AgNPs, the AgNPs nanocarriers (PEGylated), and the SBA-bioconjugated AgNPs nanocarriers [18]. This study concluded that bioconjugated AgNPs had less cytotoxic effects against non-cancerous cells than free AgNPs and had increased cytotoxicity in cancer cell lines [18].

##### Magnetic Nanoparticles

The most common, versatile, and efficient magnetic NPs being used in medical science are iron-oxide nanoparticles (IONPs). They have a strong hypointense T_2_ weighted signal (T2WI) that helps them to be detected by magnetic resonance imaging (MRI) directly [73]. They also have a biocompatible surface to which cancer specific biomolecules can be conjugated for specific targeting and to avoid nonspecific interactions [23]. These molecules are loaded on these nanocarriers without compromising their functionality. Also, the release of drugs must occur at the suitable site with a desired rate. Therapeutic agents can either be conjugated on the surface of IONPs or can be co-encapsulated along with magnetic NPs [74]. Organic polymers such as PEG, PLGA, and chitosan, etc. are used as a coating material to functionalize the surface of these nanoparticles [74]. Kaluzova et al. developed IONPs conjugated with cetuximab, which is a 152 kDa chimeric monoclonal antibody which targets the human growth factor receptor (EGFR). This bioconjugate was developed for the targeted therapy of glioblastoma (brain cancer cells). It was observed that conjugation had a more significant therapeutic effect against glioblastoma cells than individual cetuximab [23]. Currently, magnetic IONPs are being used in combination with radiotherapy for treating glioblastoma [75]. Alternating magnetic fields, applied on high concentrations of non-targeted IONPs, induce local hyperthermia, hence reducing glioblastoma cells prospectively [76]. Furthermore, clinical studies on humans have shown that IONPs have minimum toxicity and are biodegradable, which makes them an attractive clinical tool [75,77].

#### 2.1.2. Silica Nanoparticles

Silica NPs are among one of the most highly biocompatible types of nanoparticles since silica is present in most living organisms [78]. They are considered to be ‘‘Generally Recognized As Safe’’ (GRAS) by the U.S. Food and Drug Administration [79]. Silica NPs exhibit several characteristics that make them superior to other inorganic NPs [78]. These NPs have a distinctive structure whose pore and particle size can be adjustable. They have a high specific surface area and can be easily functionalized [78,80,81]. Due to their porous structure, a higher amount of drugs can be loaded and their release is time-dependent [80]. These properties make them an efficient tool for drug encapsulation [81]. Various molecules have been conjugated with silica NPs for targeting cancer cells. These include antibodies, peptides, aptamers, and drugs, etc. [80]. She et al. designed a novel strategy assisted by Eudragit to synthesize hollow mesoporous silica nanoparticles (HMSNs). They loaded 5-fluorouracil into these nanoparticles and functionalized them with epidermal growth factor (EGF) to specifically target colorectal cancer cells through EGF-EGFR interaction. The authors observed both high loading efficiency and specificity of the bioconjugates [14]. Cheng et al. conjugated polymer poly-ethylene glycol along with folic acid as a ligand (PEG−FA) on the surface of mesoporous silica NPs modified by polydopamine (PDA). They delivered doxorubicin via this novel system to cervical cancer cells [29]. Silica NPs have also been used to deliver chemotherapeutic drugs to drug-resistant cancer cell lines by conjugating them with certain ligands that target drug-resistant oncogenes such as Pgp and ABCG2, etc. [28,49].

### 2.2. Organic Nanocarriers

#### 2.2.1. Polymeric Nanoparticles

Synthetic polymers have been explored as nanomedicines since the 1940s. They are among one of the most successful first generation nanomedicines [82]. Earlier polymers such as dextrans, dextrin, and other oligosaccharides were used as coatings for other nanoparticles (e.g., iron nanoparticles) for the treatment of diseases such as anemia. From the 1970s, polymers bioconjugated with drugs and proteins, block copolymer micelles, and PEG-conjugated proteins subsequently emerged as cancer therapeutics. These can be efficiently used as drug vectors for the controlled release of drugs [82]. PEGylated proteins and aptamers have increased the number of important medical products [82,83,84]. Polylactic acid–polyethylene glycol (PLA-PEG) multiblock copolymer is of major interest regarding consideration as a drug carrier. Rigidity is provided by the PLA segments and the PEG part confers stealth behavior [85]. Due to stealth property, nanoparticles can escape from the immune system. Moreover, they can be circulated for a longer time after injection, which increases their lifespan. PEG also provides hydrophilicity to certain chemotherapeutic hydrophobic drugs, thus increasing their solubility [85]. Lupold and colleagues used a PLA-*b*-PEG copolymer to target prostate cancer by conjugating A10 RNA aptamer with the polymer. This aptamer specifically attaches to the extracellular domain of the prostate specific membrane antigen (PSMA) [86,87]. Furthermore, they encapsulated docetaxel into this bioconjugate, which increased its efficacy for prostate cancer, as is evident in in vivo studies [88,89]. Dhar et al. also targeted PSMA with aptamers and used PLGA-PEG as a nanocarrier. They encapsulated cisplatin into the nanoparticles functionalized with platinum (IV) compound *c,t,c*-[Pt(NH_3_)_2_(O_2_CCH_2_CH_2_CH_2_CH_2_CH_3_)_2_Cl_2_] (Pt IV), which acted as precursor for cisplatin. This system has been shown to lead to delivery of a lethal dose of cisplatin into cancer cells [90,91]. Additionally, tumor-specific antibodies have also been covalently linked with polymeric nanoparticles to develop target specific bioconjugates. Dhankar et al. conjugated anti-human epidermal growth factor receptor (HER-2, ErbB2) antibody with PLGA-PEG nanoparticles to target breast cancer cell [34]. Another researcher conjugated CD44 monoclonal antibody with PLGA-PEG particles encapsulated with cisplatin to target ovarian cancer cells [43]. Drugs can also be conjugated with nanoparticles instead of physically encapsulating them [92]. Conjugation stabilizes the drug, assures its delivery to cancer cells, and prevents its premature release into blood [92]. Hami et al. conjugated the anticancer drug doxorubicin with PLA-PEG polymeric micelles through the hydrazone bond. This conjugate was further decorated with a folate ligand. High in vitro cytotoxicity was observed in human ovarian cancer cells using this drug-conjugated bioconjugate compared to micelles without the folate ligand [92]. Researchers also used polymeric nanoparticles to deliver anticancer peptide cargos to the cancerous site. Kumar et al. encapsulated anticancer peptide NuBCP9 into a PEG-PLA block copolymer which induced apoptosis of cancer cells [16]. Szweda et al. conjugated met-enkephalin, an endogenous opioid therapeutic peptide, with a thermoresponsive polymer, forming polymeric mesoglobules. The targeting peptide RGD was also linked on the surface of the nanocarrier, which bound to integrin receptors overexpressed on cancer cells. However, this study focused on the utilization of the thermoresponsive polymer, and in vivo and in vitro studies are required to check its efficacy against cancer [17]. Subsequently, proteins conjugated with polymers have also been used as bioconjugates for cancer therapy. Quester et al. conjugated cytochromes P450 protein with PEG and functionalized it with folic acid to target MCF-7 breast cancer cells with low CYP activity. This enzymatic therapy activated the prodrug, tamoxifen, inside cancer cells, and hence increased the treatment efficiency and reduced the nonspecific toxicity of the drug [93]. In the presence of PEG, proteins escaped from the immune system and undesirable immunological responses associated with therapeutic protein products were also prevented [93].

Inhibitors, including anastrozole (ANS) which is an aromatase inhibitor (AI), have also been used as an anticancer medication. However, side effects, low solubility, and the short plasma half-life of inhibitors are the major limitations of inhibitor therapy. Hence, conjugation of inhibitors with polymeric nanoparticles has been shown to reduce undesirable side effects and enhanced efficiency [94]. Alyafee et al. loaded ANS into PLA-PEG-PLA nanoparticles for the treatment of breast cancer. They compared the apoptotic response of cancer cells treated with both free and loaded ANS nanoparticles (ANS NPs) and observed similar therapeutic effects [94].

Steroids are also used for cancer treatment either as a therapeutic agent (leukemia and lymphoma) or as an anti-swelling agent (glioma) [95]. β-Sitosterol (β-Sit) is a plant-based sterol which has anticancer activity against various cancer cells such as leukemia, prostate, breast, and colon cancer. Though it has low aqueous solubility and targeting efficiency, it has been found to stimulate cell apoptosis by multiple signaling pathways [96]. Andima et al. encapsulated this phytosterol into poly(lactide-co-glycolic acid) (PLGA) and poly(ethylene glycol)-block-poly(lactic acid) nanoparticles, thereby increasing its solubility and therapeutic efficacy against breast cancer [96].

#### 2.2.2. Polysaccharides

Despite having amazing properties like biodegradability and biocompatibility, synthetic polymers such as PEG, PLA, and PLGA have drawbacks like higher cost and limited loading capacity of drugs [97]. Natural polymers such as polysaccharides are nontoxic, biocompatible, and are abundantly present in nature. They are produced by algae, plants, microorganisms, and animals. Examples of polysaccharides are alginate, cellulose, dextran, hyaluronan, and chitosan, etc. They are widely used as a carrier for cancer therapeutics [98]. Dextran (Dex) has excellent aqueous solubility, biocompatibility, is easily modifiable, is widely available, and has FDA approval in parenteral formulations [36]. In several studies, cancer-specific ligands and drugs have been conjugated with dextran as a carrier. Li et al. developed a formulation by loading cisplatin into dextran and functionalizing it with the luteinizing hormone-releasing hormone (LHRH) ligand to target specific receptors in breast cancer cells. The authors observed apoptosis of cancer cells along with the positive expression of a poly ADP-ribose polymerase 1 (c-PARP1) fragment [36]. Lopalco et al. prepared nanogels from dextran and conjugated translocator protein18-kDa (TSPO) to inhibit the proliferation of glioma cell lines [99]. Recently, researchers have developed polysaccharide-doxorubicin-peptide bioconjugates for specifically targeting integrin-expressing cancer cells. They have conjugated polysaccharide carboxymethylcellulose (CMC) that had been chemically modified with doxorubicin (DOX) through amide bonds. The surface of the system was functionalized with integrin target receptor tripeptide (RGD) and L-arginine (R), a cell-penetrating amino acid. This formulation improved the chemotherapeutic efficiency by enhancing the internalization of the drug by cancer cells, synergistically targeting the integrin-rich cancer cells and minimizing the adverse effects on normal cells [37].

## 3. Combination Therapy Via Bioconjugates

### Nucleic Acid or Aptamer-Based Therapeutic Agents

Nucleic acid bioconjugates have been extensively used for detecting and treating cancers. Aptamers, single-stranded nucleic acid molecules, can be conjugated with drugs, nanocarriers, or chemotherapeutic agents. They can be used as both targeting agents and therapeutic agents. Due to the uniqueness of their structure, they can be specifically bound to cells with a high affinity via electrostatic forces, hydrogen bonding, or van der Waal’s interactions [100,101,102]. Aptamers are the ultimate in targeted therapy with respect to specificity. They are strong and versatile molecules which have amazing biomedical applications. However, they have some downlines which include degradation by nuclease in vivo and ineffective immobilization on carrier surfaces, leading to untargeted delivery. Furthermore, proper methodologies are unavailable to convert highly specific aptamer-targeted molecular recognition into detectable signals. Chemical modification and bioconjugation of aptamers with nanostructures is one of the solutions to these drawbacks [100,102]. The first aptamer to go through clinical trials for cancer treatment was AS1411. This aptamer targets nucleolin, a protein expressed in the nuclei of all cells but in the case of cancer cells overexpressed in the cytoplasm and on the plasma membrane compared to normal cells [101]. Researchers have conjugated the AS1411 aptamer to various molecules such as ^67^Ga-citrate in cobalt-ferrite nanoparticles within a silica shell matrix for radionuclide imaging. This versatile bioconjugated cancer-targeted imaging system has been observed to be used for specific cancer diagnosis and to study cellular metabolism [103]. AS1411 was also conjugated with the chemotherapeutic agent doxorubicin to form a synthetic drug-DNA adduct (DDA) to target hepatocellular carcinoma cells. Authors evaluated the efficiency of this bioconjugate in vitro and in vivo. They concluded that the bioconjugated drug showed less anticancer efficacy than the free drug; however, side effects were observed less in the case of the bioconjugates [30]. Guo et al. conjugated AS1411 with PEG-PLGA nanoparticles encapsulated with paclitaxel to enhance the anti-glioma efficacy of the drug [33]. Malignant brain tumor is difficult to treat because of the nonspecificity of drugs, and hence, targeted delivery of drugs through aptamers increases their specificity [33]. Recently, Tao et al. conjugated the AS1411 aptamer with docetaxel-loaded copolymeric nanoparticles to target breast cancer cell lines in vivo [20]. Prostate-specific membrane antigen, a surface protein expressed in healthy prostates, prostate cancer, and the vasculature of various solid tumors, is also one of the best tumor markers for imaging and therapy [101]. Lupold et al. demonstrated that the aptamers A9 and A10 specifically bind to prostate cancer cells via PSMA [86]. Wang et al. developed superparamagnetic iron oxide nanoparticles (SPIONs)–A10 aptamer bioconjugates as a theranostic tool to deliver doxorubicin to prostate cancer cells [24]. Furthermore, Jalalian et al. extended this method to other cancer cell lines and conjugated the 5TR1 aptamer (Apt), which targets mucin-1 (MUC-1) glycoform, with epirubicin-loaded SPIONs to target murine colon cancer cells (C26 cells) [31]. Another aptamer molecule, DM1 (a maytansine-derived high-potential cytotoxic agent) was conjugated with mesoporous silica nanoparticles (MSNs) that bind to epithelial cell adhesion molecules (EpCAMs). This conjugate was found to target tumors of epithelial origin such as colorectal adenocarcinoma [104]. Recently, an electrochemical sandwich biosensor was developed by conjugating two aptamers which targeted the MUC1 biomarker of MCF7 cell lines. The first aptamer (MUC1) acted as a capture aptamer that specifically bound to MCF-7 cells which had been introduced as a sample to be detected. The second aptamer, labelled with silver nanoparticles, acted as a detection aptamer which bound to captured cancer cells, forming a sandwich. This biosensor was found to be useful to identify breast cancer in the initial stages [105].

Researchers have also delivered microRNAs (miRNAs) as a therapeutic agent. They are small-sized (~22 nucleotides) noncoding RNAs that can control post-transcriptional gene expression via RNA silencing. However, RNAs are prone to degradation by nucleases present in serum and can also activate immunogenic and inflammatory responses; hence, they need to be delivered in conjugation with other molecules. Perepelyuk et al. synthesized an aptamer-hybrid nanoparticle bioconjugate delivery system which consisted of miRNA-29b as a therapeutic agent (tumor-suppressant miRNA) and MUC1 aptamer as a targeting agent. They made this nanoformulation by encapsulating miRNA-29b in human IgG and coating the nanoparticle with poloxamer-188. Copolymer poloxamer-188 provided stealth behavior to the nanoparticles [19]. The MUC1 aptamer was found to bind to the transmembrane protein, mucin, which is expressed on the surface of cancerous cells. This nucleic acid bioconjugate was used to downregulate oncoproteins DNA methyltransferase 3B (DNMT3b) and myeloid cell leukemia sequence 1 (MCL1) in A549 cells and thereby inhibit cancer cell proliferation. This bioconjugate induces cell apoptosis and prevents methylation of cancer suppressor genes [19].

siRNA therapy is also a gene silencing therapy useful for cancer treatment. Scientists have linked antibodies and ligands chemically with siRNA nanoparticles; this approach is known as the siRNA-mediated silencing (RNAi) of genes. siRNAs also target oncogenes that lead to tumor proliferation, metastasis, angiogenesis, and multidrug resistance, and inhibit apoptosis [106]. Tietze et al. utilized dendrimer as a siRNA carrier linked with EGFRvIII antibody. This antibody is the ligand most frequently used to target EGFR. This bioconjugated system was found to be highly specific, with great stability [106]. Misra et al. used the ‘nuclein’ type nanoparticle “siNozyme” from the nano-assembly of pamitoyl-bioconjugated acetyl co-enzyme-A. This system stably incorporated chemotherapeutics and biologics to melanoma cancer sites for inhibiting their growth. They targeted transcriptional gene cMyc with siRNA and used siNozyme as a carrier [107]. Recently, Shah et al. conjugated siRNA with a series of saturated and unsaturated fatty acids (palmitic acid). This bioconjugation improved the cellular uptake of siRNA, which targets oncogenic glucose regulated proteins (GRPs) and downregulates them, thus improving cancer gene therapy [32].

mRNA-based therapeutic approaches have also emerged in the past few years. Earlier, mRNAs were not popular as therapeutic agents because of their instability, immunogenicity, poor delivery mechanism, and high production cost. However, within the past several years, researchers have gained knowledge about mRNA delivery systems and have reduced their production cost. Lipids, polymers, proteins, and gold nanoparticles, etc. are some of the examples of delivery agents which have been evolved over the years [108]. Oberli et al. developed lipid nanoparticles to deliver an mRNA vaccine for cancer immunotherapy. In this study, mRNA was delivered to the cytosol of antigen-presenting immune cells to induce a cytotoxic CD 8 T-cell response. This delivery system successfully protected the mRNA from endonucleases and delivered it to the targeted cells of a B16F10 tumor animal model without damaging normal cells [109]. In another study, a nanomicelle of a PEG block copolymer attached to cholesterol at one terminal was used as a delivery carrier for mRNA to impede pancreatic tumor tissue growth [110]. Gold nanoparticles were also used to deliver mRNA to cancer cells. Yeom et al. bioconjugated *BAX* mRNA on gold nanoparticle-DNA oligonucleotide conjugates to deliver mRNA into a xenograft tumor model. *BAX* mRNA was found to synthesize BAX protein, which inhibits tumor growth by apoptosis. This gold-nanoparticle-based delivery system was found to be stable, safe, and effective in vivo [111]. 

## 4. Nanotoxicity of Nanocarriers Used in Bioconjugates

Toxicity is a foremost issue while dealing with bioconjugates and their nano-sized carriers before considering them for biomedical purposes. In most of the references considered, it is observed that the nanocarriers chosen for drug delivery are biocompatible. Moreover, conjugating them with targeting molecules further reduces their toxicity towards normal cells and increases their efficiency. Pimentel et al. have reported that conjugation of silver nanoparticles with soybean agglutinin reduces cytotoxicity in non-cancerous cells (MCF 10A) [18]. Similarly, Azizi et al. compared the cytotoxic effect of silver nanoparticles and albumin-conjugated silver nanoparticles on normal cells (MCF-10A, WBCs) and different cancerous cell lines (MCF-7, MDA-MB-231). They reported that silver nanoparticles had less cytotoxic effects against normal cell lines compared to cancer cells. Furthermore, albumin encapsulation increased the cellular uptake of nanoparticles in cancerous cells due to specific targeting of albumin on tumor cells. Hence, cytotoxicity was further reduced in non-cancerous cells after bioconjugation [112]. In another study, superparamagnetic iron oxide nanoparticles were surface modified by PEG. Cytotoxicity was reduced above 100% in fibroblasts compared to uncoated nanoparticles after being coated with PEG [113]. Hence, these particles can be used for targeted drug delivery for cancer therapy. Gold nanoparticles, which are widely used nanocarriers for drug delivery or imaging purposes, are also biocompatible and possess less cytotoxicity against normal cell lines. Mioc et al. conjugated PEG-coated gold nanoparticles with betulin, which is a pentacyclic triterpene with anti-tumor properties. They reported low cytotoxicity of PEG-coated nanoformulations in human non-melanoma cells (1BR3, HaCaT). Hence, this nanobioconjugate can be considered a safe nanocarrier [114]. Additionally, antibody-drug conjugation, which is one of the predominant therapeutic approaches against cancer cells, also manifests comparatively less toxicity. Curado et al. bioconjugated gold (I) compounds with the monoclonal antibody trastuzumab for treating HER-2 positive breast cancer cells. They observed less cytotoxicity in a non-cancerous line (MCF-10A) compared to the MCF-7 cell line [115]. However, in most of the cases the cytotoxicity assay was performed in vitro only. These in vitro studies should be extrapolated to in vivo models for establishing bioconjugates as potential cancer therapeutics. In the long term, in vivo toxicity assays must be performed for confirmation of their non-toxicity. Li et al. developed conjugated mesoporous silica nanoparticles loaded with miRNA (miR328) and surface functionalized with dopamine, PEG, epithelial cell adhesion molecule aptamer, and bevacizumab. This system was developed for dual-targeted treatment of colorectal cancer. They observed nanotoxicity of the bioconjugates both in vitro and in vivo. They reported comparatively less cytotoxicity in a normal cell line (NCM460) than a colorectal cancer cell line (SW480). Also, no systemic cytotoxicity was observed in mice treated with these nanoparticles, and hence, the bioconjugates may be considered biocompatible [116]. Only a few of the bioconjugates passed for clinical trials due to stability issues. As it is a new field, much research is to be done. 

## 5. Fate of Newly Developed Bioconjugates

Developing new anticancer bioconjugates is no doubt a revolutionary way to combat cancer. However, their synthesis will be useful only when they have successfully passed all the steps to be commercialized for treating cancer patients. These steps include in vitro studies, animal studies, and clinical trials. Only a few of the anticancer bioconjugates are being clinically passed. As mentioned in Table 1, few researchers have done animal studies. Most of the studies are done up to in vitro analysis only. In vitro cell culture shows different behavior compared to the corresponding cell type in an organism. Zellmer et al. observed that for primary murine hepatocytes, when isolated from their normal microenvironment, various genes are down- and upregulated [117]. In vitro assays are not enough to prove efficacies of new formulations [118]. Hence, in vivo animal studies are a must before clinical trials in the present scenario. Recently, cetuximab-loaded polymeric nanoparticles were approved for clinical trials phase 1. These nanoparticles were functionalized with a somatostatin analogue for the treatment of colon cancer (ClinicalTrials.gov Identifier: NCT03774680). In 2017, besponsa (inotuzumab ozogamicin) was approved by the FDA as a breakthrough therapy, it being an antibody-drug conjugate that targets CD22 of acute lymphoblastic leukemia. It is a conjugate of a monoclonal antibody that has specificity for CD22 and an anticancer drug of the calicheamicins class [119,120]. Another drug antibody conjugate, mylotarg (gemtuzumab ozogamicin), which has been developed for the treatment of acute myeloid leukemia (AML), was also approved by the FDA in 2017. This conjugate, comprised of a monoclonal antibody targeted against CD33, conjugated with cytotoxic antibiotic calicheamicin [121]. In 2013, the FDA licensed ado-trastuzumab emtansine (KADCYLA, Genentech, Inc.), which is also a drug antibody conjugate which targets HER-2 positive metastatic breast cancer. It consists of trastuzumab, a recombinant humanized monoclonal antibody which has an affinity for human epidermal growth factor receptor-2 (HER-2) overexpressed in breast cancer. The cytotoxic agent in this conjugate is the microtubule inhibitor DM1, a maytansine derivative [122]. In 2019, this conjugate was FDA-approved for HER-2 positive early breast cancer as reported on the U.S. Food and Drug Administration website. Only few conjugates have gone through clinical trials and have been approved by FDA for cancer treatment. Bioconjugation is a new field for treating cancer and needs to be further explored for its application in cancer research.

## 6. Conclusion and Future Perspectives

All studies presented here show the potential of bioconjugated nanoparticles to control tumor cells. After bioconjugation, the therapeutic effect of anticancer agents becomes more significant when compared to their individual effects. Trends in the application of various bioconjugates in cancer research clearly show that they can be potential tools for cancer diagnosis and treatment. There is scope for developing better bioconjugates with improved therapeutic efficacy, target specificity, multimodality, and signal intensity. However, as of now, most of the studies have been carried out in vitro. In addition to in vitro studies, animal model in vivo studies are a prerequisite to understanding the full potential of bioconjugates. Presently, bioconjugates are still at a proof-of-concept stage and cannot be immediately introduced in the clinical stage. Efficacy of in vitro results often does not translate to the clinical stage. Potential drugs which show significant reduction in cancer cell growth in in vitro models and/or reduction in tumor size in animal models might not give desirable survival rates during clinical trials. We need to carry out a substantial number of fundamental studies to enhance their stability, target specificity, and efficiency. These concerns must be addressed thoroughly before approving these molecules as drugs and utilizing them for the benefits of humans. An additional challenge faced by these bioconjugates is their production cost. The production cost of bioconjugates is increased when therapeutic biomolecules such as antibodies, aptamers, nucleic acids, and peptides etc. are conjugated with them. Hence, in the present scenario, it seems practically unsustainable to provide this therapy to common people and in underdeveloped countries. At the ground level, hospitals are still using chemotherapy for treatment. More and more studies and government assistance is required to bring these potent therapeutics to ground level. Undoubtedly, researchers will continue to explore these exciting developments and bring bioconjugates to the clinical and commercial level so as to overcome the shortcomings of present available techniques.

## Figures and Tables

**Table 1:** Some examples of different bioconjugates developed so far. (IONPs: Iron oxide nanoparticles, EGFR: Epidermal growth factor receptor, cRGD: cyclic arginine-glycine-aspartate motif, LHRH: Luteinizing hormone-releasing hormone, HER2: human epidermal growth factor receptor 2, 7\NPs: nanoparticles, QD: quantum dots, MDR: multidrug resistance, MUC1: mucin 1, Pgp: P-glycoprotein, PLGA: poly lactic-co-glycolic acid, TPGS: tocopheryl polyethylene glycol succinate, HA: Hyaluronic acid).

S No.	Carrier	Therapeutic Agent	Ligand	Targeted Site	Mechanism of Action	Study Model	Ref.
1	IONPs	Cituximab	Cituximab/EGFRvIIIAb	EGFR glioblastoma	Caspase-3 activation, apoptosis	In vitro and in vivo studies	[23]
2	SPIONS	Doxorubicin	A10 RNA Aptamer	Prostate-specific membrane antigen of prostate cancer cells	Caspase-3 activation, apoptosis	In vitro	[24]
3	SPIONS	Doxorubicin	cRGD peptide	Integrin positive U87MG cell lines	Caspase-3 activation, apoptosis	In vitro	[25]
4	Magnetic Fe-Zn NPs	Doxorubicin	LHRH	LHRH-expressing breast cancer cells	Caspase-3 activation, apoptosis	In vitro	[15]
5	Gold NPs	relAsiRNA	Transferrin targeting ligand (Tf)/folic acid (FA)	Transferrin-receptor- and folate-receptor- expressing prostate cancer	Downregulates relA gene (protooncogene)	In vitro	[26]
6	Gold nanospheres	Radiation therapy	Anti-c-Met antibody	Cervical cancer	Fas signaling pathway (apoptosis)	In vitro	[27]
7	Silver NPs	Soybean agglutinin	Soybean agglutinin	N-acetylgalactosamine and β-D galactose (breast cancer cells)	Autophagy, apoptosis, DNA damage	In vitro	[18]
8	Silica NPs	5-Fluorouracil	Epidermal growth factor	Epidermal-growth-factor-receptor-expressing colorectal cancer cells	Inhibits thymidylate synthase, causes thymineless death	In vitro	[14]
9	Silica NPs	Doxorubicin	Pgp siRNA	Knockdown pgp gene, multidrug-resistant KBV1 cell line	Caspase-3 activation, apoptosis	In vitro	[28]
10	Silica NPs	Doxorubicin	PEG-folic acid	Folate-expressing cervical cancer cells	Caspase-3 activation, apoptosis	In vitro and in vivo	[29]
11	_	IFNα2a	Hyaluranan acid	CD44+ ovarian cancer cells	JAK-STAT pathway, apoptosis	In vitro and in vivo	[21]
12	Aptamer	miRNA 29b	MUC1 aptamer	MUC1 transmembrane protein, lung cancer	Downregulates antiapoptotic proteins MCL1 and DNMT3B	In vitro	[19]
13	Aptamer	Doxorubicin	AS1411	Nucleolin present on membrane of hepatocellular carcinoma	Apoptosis	In vitro and in vivo	[30]
14	SPIONs	Epirubicin	5TR1 aptamer	Mucin1-glycoprotein-expressing colon cancer cell lines (C26)	Topoisomerase inhibitor	In vitro and in vivo	[31]
15	Fatty acid	siRNAs	siRNAs	Oncogenic glucose-regulated proteins (GRPs) in prostate cancer cells (PC-3)	siRNAs downregulates GRPs, apoptosis	In vitro	[32]
16	PLGA-TPGS NPs	Docitaxel	AS1411 aptamer	Nucleolin present on membrane of adenocarcinoma	Inhibition of mitotic cell division between metaphase and anaphase, blocks bcl2 oncoprotein, apoptosis	In vitro and in vivo	[20]
17	Thermoresponsive polymers	Met-enkaphalin peptide	RGD targeting ligand	RGD peptide	Halt growth of cells by immunological mechanisms	-	[17]
18	PLGA-PEG	Paclitaxel	AS1411	Nucleolin expressed on C6 glioma cells	Inhibition of mitotic cell division between metaphase and anaphase, blocks bcl-2 oncoprotein, apoptosis	In vitro and in vivo	[33]
19	PLGA-PEG	Docetaxel	HER-2 Ab	HER-2-expressing breast cancer cells	Inhibition of mitotic cell division between metaphase and anaphase, prevents microtubule depolymerization, apoptosis	In vitro	[34]
20	PLA-PEG	NuBCP9 peptide	Bcl-2	MCF-7 breast cancer and HepG2 hepatocellular carcinoma cells	Bcl-2-conversion-dependent apoptosis	In vitro and in vivo	[16]
21	Pullalan	Paclitaxel	Alendronate (ALN)	Hydroxyapatite, breast cancer bone metastasis	Inhibition of mitotic cell division between metaphase and anaphase, blocks bcl-2 oncoprotein, apoptosis	In vitro	[35]
22	Dextran	Cisplatin	LHRH-targeting ligand	LHRH receptors on breast cancer cells	Caspase-3- and caspase-7-activated apoptosis	In vitro and in vivo	[36]
23	Polysaccharide (carboxymethyl cellulose)	Doxorubicin	Integrin target receptor tripeptide (RGD), L-arginine	Integrin-expressing HEK293t cell lines	Apoptosis	In vitro and in vivo	[37]
24	Virus-like NPs (tHBcAg)	Doxorubicin	Folic acid	Folate-expressing HeLa cells	Caspase-3 activation, apoptosis	In vitro	[38]
25	_	HA-cytarabine (Ara-C)	Folic acid	Folate-expressing leukemia cancer cells	Inhibition of DNA polymerase, apoptosis	In vitro	[39]
26	_	Methotrexate	(D-Lys^6^)- LHRH	LHRH-expressing prostate cancer cells	Apoptosis	In vitro and in vivo	[40]
27	CdSe/ZnS core/shell QD	Doxorubicin	JB434 (cell uptake peptide)	H69AR (human small cell lung carcinoma)	JB434 allows QDs to penetrate MDR cancer cells, doxorubicin leads to apoptosis	In vitro	[41]
28	Graphene QD	Biosurfactant	Folic acid	Folate-expressing breast cancer cell lines (MCF-7)	Not determined	In vitro	[42]

## References

[1] All Cancers Fact Sheet, GLOBOCAN 2018. The Global Cancer Observatory. https://gco.iarc.fr/today/data/factsheets/cancers/39-All-cancers-fact-sheet.pdf.

[2] Bray F., Ferlay J., Soerjomataram I., Siegel R.L., Torre L.A., Jemal A. (2018). Global Cancer Statistics 2018: GLOBOCAN Estimates of Incidence and Mortality Worldwide for 36 Cancers in 185 Countries. CA Cancer J. Clin..

[3] Arruebo M., Vilaboa N., Sáez-Gutierrez B., Lambea J., Tres A., Valladares M., González-Fernández Á. (2011). Assessment of the evolution of cancer treatment therapies. Cancers.

[4] Cameron F., Whiteside G., Perry C. (2011). Ipilimumab: First global approval. Drugs.

[5] Griffin A.M., Butow P.N., Coates A.S., Childs A.M., Ellis P.M., Dunn S.M., Tattersall M.H.N. (1996). On the receiving end V: Patient perceptions of the side effects of cancer chemotherapy in 1993. Ann. Oncol..

[6] Remesh A. (2012). Toxicities of anticancer drugs and its management. Int. J. Basic Clin. Pharmacol..

[7] Luo J., Solimini N.L., Elledge S.J. (2009). Principles of Cancer Therapy: Oncogene and Non-oncogene Addiction. Cell.

[8] Sinha R. (2006). Nanotechnology in cancer therapeutics: Bioconjugated nanoparticles for drug delivery. Mol. Cancer Ther..

[9] Salmaso S., Caliceti P. (2011). Chapter 11—Peptide and Protein Bioconjugation: A Useful Tool to Improve the Biological Performance of Biotech Drugs.

[10] Werengowska-CieTwierz K., Wisniewski M., Terzyk A.P., Furmaniak S. (2015). The Chemistry of Bioconjugation in Nanoparticles-Based Drug Delivery System. Adv. Condens. Matter Phys..

[11] Du W., Yuan Y., Wang L., Cui Y., Wang H., Xu H., Liang G. (2015). Multifunctional Bioconjugate for Cancer Cell-Targeted Theranostics. Bioconjugate Chem..

[12] Hermanson G.T. (2013). Chapter 1—Introduction to Bioconjugation. Bioconjugate Tech..

[13] Fernandes C.S.M., Teixeira G.D.G., Iranzo O., Roque A.C.A. (2018). Chapter 5—Engineered Protein Variants for Bioconjugation. Biomedical Applications of Functionalized Nanomaterials.

[14] She X., Chen L., Velleman L., Li C., Zhu H., He C., Wang T., Shigdar S., Duan W., Kong L. (2015). Fabrication of high specificity hollow mesoporous silica nanoparticles assisted by Eudragit for targeted drug delivery. J. Colloid Interface Sci..

[15] Varshosaz J., Hassanzadeh F., Aliabadi H.S., Khoraskani F.R., Mirian M., Behdadfar B. (2016). Targeted delivery of doxorubicin to breast cancer cells by magnetic LHRH chitosan bioconjugated nanoparticles. Int. J. Biol. Macromol..

[16] Kumar M., Gupta D., Singh G., Sharma S., Bhat M., Prashant C.K., Dinda A.K., Kharbanda S., Kufe D., Singh H. (2014). Novel polymeric nanoparticles for intracellular delivery of peptide cargos: Antitumor efficacy of the BCL-2 conversion peptide NuBCP-9. Cancer Res..

[17] Szweda R., Trzebicka B., Dworak A., Otulakowski L., Kosowski D., Hertlein J., Haladjova E., Rangelov S., Szweda D. (2016). Smart Polymeric Nanocarriers of Met-enkephalin. Biomacromolecules.

[18] Pimentel R.G.C., Robles Botero V., Martínez E.S.M., Gómez García C., Hinestroza J.P. (2016). Soybean agglutinin-conjugated silver nanoparticles nanocarriers in the treatment of breast cancer cells. J. Biomater. Sci. Polym. Ed..

[19] Perepelyuk M., Maher C., Lakshmikuttyamma A., Shoyele S.A. (2016). Aptamer-hybrid nanoparticle bioconjugate efficiently delivers miRNA-29b to non-small-cell lung cancer cells and inhibits growth by downregulating essential oncoproteins. Int. J. Nanomed..

[20] Tao W., Zeng X., Wu J., Zhu X., Yu X., Zhang X., Zhang J., Liu G., Mei L. (2016). Polydopamine-based surface modification of novel nanoparticle-aptamer bioconjugates for in vivo breast cancer targeting and enhanced therapeutic effects. Theranostics.

[21] Montagner I.M., Merlo A., Carpanese D., Dalla Pietà A., Mero A., Grigoletto A., Loregian A., Renier D., Campisi M., Zanovello P. (2016). A site-selective hyaluronan-interferonα2a conjugate for the treatment of ovarian cancer. J. Control. Release.

[22] Sun X., Zhang G., Patel D., Stephens D., Gobin A.M. (2012). Targeted cancer therapy by immunoconjugated gold-gold sulfide nanoparticles using protein g as a cofactor. Ann. Biomed. Eng..

[23] Kaluzova M., Bouras A., Machaidze R., Hadjipanayis C.G. (2015). Targeted therapy of glioblastoma stem-like cells and tumor non-stem cells using cetuximab-conjugated iron-oxide nanoparticles. Oncotarget.

[24] Wang A.Z., Bagalkot V., Vasilliou C.C., Gu F., Alexis F., Zhang L., Shaikh M., Yuet K., Cima M.J., Langer R. (2008). Superparamagnetic Iron Oxide Nanoparticle–Aptamer Bioconjugates for Combined Prostate Cancer Imaging and Therapy. ChemMedChem.

[25] Yu M.K., Park J., Jeong Y.Y., Moon W.K., Jon S. (2010). Integrin-targeting thermally cross-linked superparamagnetic iron oxide nanoparticles for combined cancer imaging and drug delivery. Nanotechnology.

[26] Guo J., O’Driscoll C.M., Holmes J.D., Rahme K. (2016). Bioconjugated gold nanoparticles enhance cellular uptake: A proof of concept study for siRNA delivery in prostate cancer cells. Int. J. Pharm..

[27] Liang Y., Liu J., Liu T., Yang X. (2017). Anti-c-met antibody bioconjugated with hollow gold nanospheres as a novel nanomaterial for targeted radiation ablation of human cervical cancer cell. Oncol. Lett..

[28] Meng H., Liong M., Xia T., Li Z., Ji Z., Zink J.I., Nel A.E. (2010). Engineered Design of Mesoporous Silica Nanoparticles to Deliver Doxorubicin and Pgp siRNA to Overcome Drug Resistance in a Cancer Cell Line. ACS Nano.

[29] Cheng W., Nie J., Xu L., Liang C., Peng Y., Liu G., Wang T., Mei L., Huang L., Zeng X. (2017). pH-Sensitive Delivery Vehicle Based on Folic Acid-Conjugated Polydopamine-Modified Mesoporous Silica Nanoparticles for Targeted Cancer Therapy. ACS Appl. Mater. Interfaces.

[30] Le Trinh T., Zhu G., Xiao X., Puszyk W., Sefah K., Wu Q., Tan W., Liu C. (2015). A Synthetic Aptamer-Drug Adduct for Targeted Liver Cancer Therapy. PLoS ONE.

[31] Jalalian S.H., Taghdisi S.M., Hamedani N.S., Kalat S.A.M., Lavaee P., ZandKarimi M., Ghows N., Jaafari M.R., Naghibi S., Danesh N.M. (2013). Epirubicin loaded super paramagnetic iron oxide nanoparticle-aptamer bioconjugate for combined colon cancer therapy and imaging in vivo. Eur. J. Pharm. Sci..

[32] Shah S.S., Cultrara C.N., Kozuch S.D., Patel M.R., Ramos J.A., Samuni U., Zilberberg J., Sabatino D. (2018). Direct Transfection of Fatty Acid Conjugated siRNAs and Knockdown of the Glucose-Regulated Chaperones in Prostate Cancer Cells. Bioconjug. Chem..

[33] Guo J., Gao X., Su L., Xia H., Gu G., Pang Z., Jiang X., Yao L., Chen J., Chen H. (2011). Aptamer-functionalized PEG-PLGA nanoparticles for enhanced anti-glioma drug delivery. Biomaterials.

[34] Dhankar R., Jain A.K., Arora S., Rath G., Goyal A.K., Rathee P., Kumar M.S., Saxena A.K., Sharma P.R., Kumar S. (2011). HER-2 targeted immunonanoparticles for breast cancer chemotherapy. J. Appl. Pharm. Sci..

[35] Bonzi G., Salmaso S., Scomparin A., Eldar-Boock A., Satchi-Fainaro R., Caliceti P. (2015). Novel Pullulan Bioconjugate for Selective Breast Cancer Bone Metastases Treatment. Bioconjug. Chem..

[36] Li M., Tang Z., Zhang Y., Lv S., Li Q., Chen X. (2015). Targeted delivery of cisplatin by LHRH-peptide conjugated dextran nanoparticles suppresses breast cancer growth and metastasis. Acta Biomater..

[37] Mansur A.A.P., Carvalho S.M., Lobato Z.I.P., Leite M.D.F., Armando da Silva Cunha J., Mansur H.S. (2018). Design and Development of Polysaccharide-Doxorubicin-Peptide Bioconjugates for Dual Synergistic E ff ects of Integrin-Targeted and Cell-Penetrating Peptides for Cancer Chemotherapy. Bioconjug. Chem..

[38] Biabanikhankahdani R., Ho K., Alitheen N., Tan W. (2018). A Dual Bioconjugated Virus-Like Nanoparticle as a Drug Delivery System and Comparison with a pH-Responsive Delivery System. Nanomaterials.

[39] Liu J., Zhao D., He W., Zhang H., Li Z., Luan Y. (2017). Nanoassemblies from amphiphilic cytarabine prodrug for leukemia targeted therapy. J. Colloid Interface Sci..

[40] Zhu S., Wang Q., Jiang J., Luo Y., Sun Z. (2016). A conjugate of methotrexate and an analog of luteinizing hormone releasing hormone shows increased efficacy against prostate cancer. Sci. Rep..

[41] Sangtani A., Petryayeva E., Susumu K., Oh E., Huston A.L., Lasarte-aragones G., Medintz I.L., Algar W.R., Delehanty J.B. (2019). Nanoparticle-Peptide-Drug Bioconjugates for Unassisted Defeat of Multidrug Resistance in a Model Cancer Cell Line. Bioconjug. Chem..

[42] Bansal S., Singh J., Kumari U., Kaur I.P., Barnwal R.P., Kumar R., Singh S., Singh G., Chatterjee M. (2019). Development of biosurfactant-based graphene quantum dot conjugate as a novel and fluorescent theranostic tool for cancer. Int. J. Nanomed..

[43] Bai M.Y., Liu S.Z. (2014). A simple and general method for preparing antibody-PEG-PLGA sub-micron particles using electrospray technique: An in vitro study of targeted delivery of cisplatin to ovarian cancer cells. Colloids Surf. B Biointerfaces.

[44] Schuster S., Biri-Kovács B., Szeder B., Buday L., Gardi J., Szabó Z., Halmos G., Mezo G. (2018). Enhanced In Vitro Antitumor Activity of GnRH-III-Daunorubicin Bioconjugates Influenced by Sequence Modification. Pharmaceutics.

[45] Gottesman M.M., Fojo T., Bates S.E. (2002). Multidrug Resistance in Cancer: Role of Atp-Dependent Transporters. Nat. Rev. Cancer.

[46] Thomas H., Coley H.M. (2003). Overcoming Multidrug Resistance in Cancer: An Update on the Clinical Strategy of Inhibiting P-Glycoprotein. Cancer Control.

[47] Lelle M., Freidel C., Kaloyanova S., Tabujew I., Schramm A., Musheev M., Niehrs C., Müllen K., Peneva K. (2017). Overcoming drug resistance by cell-penetrating peptide-mediated delivery of a doxorubicin dimer with high DNA-binding affinity. Eur. J. Med. Chem..

[48] Szakács G., Paterson J.K., Ludwig J.A., Booth-Genthe C., Gottesman M.M. (2006). Targeting multidrug resistance in cancer. Nat. Rev. Drug Discov..

[49] Qi X., Yu D., Jia B., Jin C., Liu X., Zhao X., Zhang G. (2016). Targeting CD133 + laryngeal carcinoma cells with chemotherapeutic drugs and siRNA against ABCG2 mediated by thermo/pH-sensitive mesoporous silica nanoparticles. Tumor Biol..

[50] Sangtani A., Petryayeva E., Wu M., Susumu K., Oh E., Huston A.L., Lasarte-aragones G., Medintz I.L., Algar W.R., Delehanty J.B. (2018). Intracellularly Actuated Quantum Dot-Peptide-Doxorubicin Nanobioconjugates for Controlled Drug Delivery via the Endocytic Pathway. Bioconjug. Chem..

[51] Postow M.A., Callahan M.K., Wolchok J.D. (2015). Immune checkpoint blockade in cancer therapy. J. Clin. Oncol..

[52] Pardoll D.M. (2016). The blockade of immune checkpoints in cancer immunotherapy. Nat. Rev. Cancer.

[53] Larkin J., Chiarion-Sileni V., Gonzalez R., Grob J.J., Cowey C.L., Lao C.D., Schadendorf D., Dummer R., Smylie M., Rutkowski P. (2017). Combined Nivolumab and Ipilimumab or Monotherapy in Previously Untreated Melanoma. N. Engl. J. Med..

[54] Ishihara J., Fukunaga K., Ishihara A., Larsson H.M., Potin L., Hosseinchi P., Galliverti G., Swartz M.A., Hubbell J.A. (2017). Matrix-binding checkpoint immunotherapies enhance antitumor efficacy and reduce adverse events. Sci. Transl. Med..

[55] Ishihara J., Ishihara A., Sasaki K., Lee S.S.-Y., Williford J.M., Yasui M., Abe H., Potin L., Hosseinchi P., Fukunaga K. (2019). Targeted antibody and cytokine cancer immunotherapies through collagen affinity. Sci. Transl. Med..

[56] Gong T., Olivo M., Dinish U.S., Goh D., Kong K.V., Yong K.T. (2013). Engineering bioconjugated gold nanospheres and gold nanorods as label-free plasmon scattering probes for ultrasensitive multiplex dark-field imaging of cancer cells. J. Biomed. Nanotechnol..

[57] Singh M., Chandrasekaran N., Mukherjee A., Kumar M., Kumaraguru A.K. (2014). Cancerous cell targeting and destruction using pH stabilized amperometric bioconjugated gold nanoparticles from marine macroalgae, Padina gymnospora. Bioprocess Biosyst. Eng..

[58] Zhu J., Yong K.T., Roy I., Hu R., Ding H., Zhao L., Swihart M.T., He G.S., Cui Y., Prasad P.N. (2010). Additive controlled synthesis of gold nanorods (GNRs) for two-photon luminescence imaging of cancer cells. Nanotechnology.

[59] Fixler D., Ankri R., Kaplan I., Novikov I., Hirshberg A. (2014). Diffusion reflection: A novel method for detection of oral cancer. J. Dent. Res..

[60] Bickford L.R., Agollah G., Drezek R., Yu T.K. (2010). Silica-gold nanoshells as potential intraoperative molecular probes for HER2-overexpression in ex vivo breast tissue using near-infrared reflectance confocal microscopy. Breast Cancer Res. Treat..

[61] Retnakumari A., Setua S., Menon D., Ravindran P., Muhammed H., Pradeep T., Nair S., Koyakutty M. (2010). Molecular-receptor-specific, non-toxic, near-infrared-emitting Au cluster-protein nanoconjugates for targeted cancer imaging. Nanotechnology.

[62] Leung K. (2011). Poly(Ethylene Glycol)- Coated Gold Nanocages Bioconjugated with [Nle4,D-Phe7]-α-Melanotropin-Stimulating Hormone. https://www.ncbi.nlm.nih.gov/pubmed/21755635.

[63] Day E.S., Bickford L.R., Slater J.H., Riggall N.S., Drezek R.A., West J.L. (2010). Antibody-conjugated gold-gold sulfide nanoparticles as multifunctional agents for imaging and therapy of breast cancer. Int. J. Nanomed..

[64] Rana S., Yeh Y.-C., Rotello V.M. (2010). Engineering the Nanoparticle-Protein Interface: Applications and Possibilities. Curr. Opin. Chem. Biol..

[65] Mocan L., Matea C., Tabaran F.A., Mosteanu O., Pop T., Puia C., Agoston-Coldea L., Zaharie G., Mocan T., Buzoianu A.D. (2017). Selective ex vivo photothermal nano-therapy of solid liver tumors mediated by albumin conjugated gold nanoparticles. Biomaterials.

[66] Diem P.H.N., Thao D.T.T., Van Phu D., Duy N.N., Quy H.T.D., Hoa T.T., Hien N.Q. (2017). Synthesis of Gold Nanoparticles Stabilized in Dextran Solution by Gamma Co-60 Ray Irradiation and Preparation of Gold Nanoparticles/Dextran Powder. J. Chem..

[67] Spadavecchia J., Movia D., Moore C., Maguire C.M., Moustaoui H., Casale S., Volkov Y., Prina-mello A. (2016). Targeted polyethylene glycol gold nanoparticles for the treatment of pancreatic cancer: From synthesis to proof-of-concept in vitro studies. Int. J. Nanomed..

[68] Liu Q., Sacco P., Marsich E., Furlani F., Arib C., Djaker N., de la Chapelle M.L., Donati I., Spadavecchia J. (2018). Lactose-Modified Chitosan Gold(III)-PEGylated Complex-Bioconjugates: From Synthesis to Interaction with Targeted Galectin-1 Protein. Bioconjug. Chem..

[69] Chen X., Schluesener H.J. (2008). Nanosilver: A nanoproduct in medical application. Toxicol. Lett..

[70] Beyene H.D., Werkneh A.A., Bezabh H.K., Ambaye T.G. (2017). Synthesis Paradigm and Applications of Silver Nanoparticles (AgNPs), A Review. Sustain. Mater. Technol..

[71] De Matteis V., Cascione M., Toma C.C., Leporatti S. (2018). Silver Nanoparticles: Synthetic Routes, In Vitro Toxicity and Theranostic Applications for Cancer Disease. Nanomaterials.

[72] Carlson C., Hussain S.M., Schrand A.M., Braydich-Stolle K.L., Hess K.L., Jones R.L., Schlager J.J. (2008). Unique Cellular Interaction of Silver Nanoparticles: Size-Dependent Generation of Reactive Oxygen Species. J. Phys. Chem. B.

[73] Choi J.S., Jun Y.W., Yeon S.I., Kim H.C., Shin J.S., Cheon J. (2006). Biocompatible heterostructured nanoparticles for multimodal biological detection. J. Am. Chem. Soc..

[74] Wahajuddin S.A. (2012). Superparamagnetic iron oxide nanoparticles: Magnetic nanoplatforms as drug carriers. Int. J. Nanomed..

[75] Maier-Hauff K., Ulrich F., Nestler D., Niehoff H., Wust P., Thiesen B., Orawa H., Budach V., Jordan A. (2011). Efficacy and safety of intratumoral thermotherapy using magnetic iron-oxide nanoparticles combined with external beam radiotherapy on patients with recurrent glioblastoma multiforme. J. Neurooncol..

[76] Bouras A., Kaluzova M., Hadjipanayis C.G. (2015). Radiosensitivity enhancement of radioresistant glioblastoma by epidermal growth factor receptor antibody-conjugated iron-oxide nanoparticles. J. Neurooncol..

[77] Mahmoudi M., Hofmann H., Rothen-Rutishauser B., Petri-Fink A. (2012). Assessing the in vitro and in vivo toxicity of superparamagnetic iron oxide nanoparticles. Chem. Rev..

[78] Caltagirone C., Bettoschi A., Garau A., Montis R. (2015). Silica-based nanoparticles: A versatile tool for the development of efficient imaging agents. Chem. Soc. Rev..

[79] US Food and Drug Administration GRAS Substances (SCOGS) Database—Select Committee on GRAS Substances (SCOGS) Opinion: Silicates. http://wayback.archive-it.org/7993/20171031063508/https://www.fda.gov/Food/IngredientsPackagingLabeling/GRAS/SCOGS/ucm260849.htm.

[80] Watermann A., Brieger J. (2017). Mesoporous Silica Nanoparticles as Drug Delivery Vehicles in Cancer. Nanomaterials.

[81] Gao Y., Chen Y., Ji X., He X., Yin Q., Zhang Z., Shi J., Li Y. (2011). Controlled Intracellular Release of Doxorubicin in Multidrug-Resistant Cancer Cells by Tuning the Shell-Pore Sizes of Mesoporous Silica Nanoparticles. Am. Chem. Soc..

[82] Duncan R., Vicent M.J. (2013). Polymer therapeutics-prospects for 21st century: The end of the beginning. Adv. Drug Deliv. Rev..

[83] Pasut G., Veronese F.M. (2012). State of the art in PEGylation: The great versatility achieved after forty years of research. J. Control. Release.

[84] Keefe A.D., Pai S., Ellington A. (2010). Aptamers as therapeutics. Nat. Rev. Drug Discov..

[85] Quesnel R., Hildgen P. (2005). Synthesis of PLA-b-PEG multiblock copolymers for stealth drug carrier preparation. Molecules.

[86] Lupold S.E., Hicke B.J., Lin Y., Coffey D.S. (2002). Identification and characterization of nuclease-stabilized RNA molecules that bind human prostate cancer cells via the prostate-specific membrane antigen. Cancer Res..

[87] Farokhzad O., Jon S., Khadelmhosseini A., Tran T., LaVan D., Langer R. (2004). Nanopartideaptamer bioconjugates: A new approach for targeting prostate cancer cells. Cancer Res..

[88] Farokhzad O., Cheng J., Teply B., Sherifi I., Jon S., Kantoff P., Richie J., Langer R. (2006). Targeted nanoparticle-aptamer bioconjugates for cancer chemotherapy in vivo. Proc. Natl. Acad. Sci. USA.

[89] Cheng J., Teply B.A., Sherifi I., Sung J., Luther G., Gu F.X., Levy-Nissenbauma E., Moreno A.F.R., Langer R., Farokhzad O.C. (2007). Formulation of Functionalized PLGA-PEG Nanoparticles for In Vivo Targeted Drug Delivery. Biomaterials.

[90] Dhar S., Gu F.X., Langer R., Farokhzad O.C., Lipparda S.J. (2008). Targeted delivery of cisplatin to prostate cancer cells by aptamer functionalized Pt(IV) prodrug-PLGA–PEG nanoparticles. Proc. Natl. Acad. Sci. USA.

[91] Dhar S., Kolishetti N., Lippard S.J., Farokhzad O.C. (2011). Targeted delivery of a cisplatin prodrug for safer and more effective prostate cancer therapy in vivo. Proc. Natl. Acad. Sci. USA.

[92] Hami Z., Amini M., Ghazi-Khansari M., Rezayat S.M., Gilani K. (2014). Doxorubicin-conjugated PLA-PEG-Folate based polymeric micelle for tumor-targeted delivery: Synthesis and in vitro evaluation. DARU J. Pharm. Sci..

[93] Quester K., Juarez-Moreno K., Secundino I., Roseinstein Y., Alejo K.P., Huerta-Saquero A., Vazquez-Duhalt R. (2017). Cytochrome P450 Bioconjugate as a Nanovehicle for Improved Chemotherapy Treatment. Macromol. Biosci..

[94] Alyafeeu Y.A., Alaamery M., Bawazeer S., Almutairi M.S., Alghamdi B., Alomran N., Sheereen A., Daghestani M., Massadeh S. (2018). Preparation of anastrozole loaded PEG-PLA nanoparticles: Evaluation of apoptotic response of breast cancer cell lines. Int. J. Nanomed..

[95] Lossignol D. (2016). A little help from steroids in oncology. J. Transl. Intern. Med..

[96] Andima M., Costabile G., Isert L., Ndakala A., Derese S., Merkel O., Andima M., Costabile G., Isert L., Ndakala A.J. (2018). Evaluation of β-Sitosterol Loaded PLGA and PEG-PLA Nanoparticles for Effective Treatment of Breast Cancer: Preparation, Physicochemical Characterization, and Antitumor Activity. Pharmaceutics.

[97] Goodarzi N., Varshochian R., Kamalinia G., Atyabi F., Dinarvand R. (2013). A review of polysaccharide cytotoxic drug conjugates for cancer therapy. Carbohydr. Polym..

[98] Basu A., Kunduru K.R., Abtew E., Domb A.J. (2015). Polysaccharide-Based Conjugates for Biomedical Applications. Bioconjug. Chem..

[99] Lopalco A., Cutrignelli A., Denora N., Perrone M., Iacobazzi R.M., Fanizza E., Lopedota A., Depalo N., De Candia M., Franco M. (2018). Delivery of proapoptotic agents in glioma cell lines by TSPO ligand–dextran nanogels. Int. J. Mol. Sci..

[100] Pednekar P.P., Jadhav K.R., Kadam V.J. (2012). Aptamer-dendrimer bioconjugate: A nanotool for therapeutics, diagnosis, and imaging. Expert Opin. Drug Deliv..

[101] Nimjee S.M., White R.R., Becker R.C., Sullenger B.A. (2017). Aptamers as Therapeutics. Annu. Rev. Pharmacol. Toxicol..

[102] Lee J.H., Yigit M.V., Mazumdar D., Lu Y. (2010). Molecular Diagnostic and Drug Delivery Agents based on Aptamer-Nanomaterial Conjugates. Adv. Drug Deliv. Rev..

[103] Hwang D.W., Ko H.Y., Lee J.H., Kang H., Ryu S.H., Song I.C., Lee D.S., Kim S. (2010). A Nucleolin-Targeted Multimodal Nanoparticle Imaging Probe for Tracking Cancer Cells Using an Aptamer. J. Nucl. Med..

[104] Li Y., Duo Y., Bao S., He L., Ling K., Luo J., Zhang Y., Huang H., Zhang H., Yu X. (2017). EpCAM aptamer-functionalized polydopamine- coated mesoporous silica nanoparticles loaded with DM1 for targeted therapy in colorectal cancer. Int. J. Nanomed..

[105] Yazdanparast S., Benvidi A., Banaei M., Nikukar H., Tezerjani M.D., Azimzadeh M. (2018). Dual-aptamer based electrochemical sandwich biosensor for MCF-7 human breast cancer cells using silver nanoparticle labels and a poly (glutamic acid)/MWNT nanocomposite. Microchim. Acta.

[106] Tietze S., Schau I., Michen S., Ennen F., Janke A., Schackert G., Aigner A., Appelhans D., Temme A. (2017). A Poly(Propyleneimine) Dendrimer-Based Polyplex-System for Single-Chain Antibody-Mediated Targeted Delivery and Cellular Uptake of SiRNA. Small.

[107] Misra S.K., Kampert T.L., Pan D. (2018). Nano-Assembly of Pamitoyl-Bioconjugated Coenzyme-A for Combinatorial Chemo-Biologics in Transcriptional Therapy. Bioconjug. Chem..

[108] Li B., Zhang X., Dong Y. (2019). Nanoscale platforms for messenger RNA delivery. Wiley Interdiscip. Rev. Nanomed. Nanobiotechnol..

[109] Oberli M.A., Reichmuth A.M., Dorkin J.R., Mitchell M.J., Fenton O.S., Jaklenec A., Anderson D.G., Langer R., Blankschtein D. (2017). Lipid Nanoparticle Assisted mRNA Delivery for Potent Cancer Immunotherapy. Nano Lett..

[110] Uchida S., Kinoh H., Ishii T., Matsui A., Tockary T.A., Takeda K.M., Uchida H., Osada K., Itaka K., Kataoka K. (2016). Systemic delivery of messenger RNA for the treatment of pancreatic cancer using polyplex nanomicelles with a cholesterol moiety. Biomaterials.

[111] Yeom J.H., Ryou S.M., Won M., Park M., Bae J., Lee K. (2013). Inhibition of Xenograft Tumor Growth by Gold Nanoparticle-DNA Oligonucleotide Conjugates-Assisted Delivery of BAX mRNA. PLoS ONE.

[112] Azizi M., Ghourchian H., Yazdian F., Bagherifam S., Bekhradnia S., Nyström B. (2017). Anti-cancerous effect of albumin coated silver nanoparticles on MDA-MB 231 human breast cancer cell line. Sci. Rep..

[113] Gupta A.K., Wells S. (2004). Surface-Modified Superparamagnetic Nanoparticles for Drug Delivery: Preparation, Characterization, and Cytotoxicity Studies. IEEE Trans. Nanobiosci..

[114] Mioc M., Pavel I.Z., Ghiulai R., Coricovac D.E., Farcas C., Mihali C., Oprean C., Serafim V., Popovici R.A., Dehelean C.A. (2018). The Cytotoxic Effects of Betulin-Conjugated Gold Nanoparticles as Stable Formulations in Normal and Melanoma Cells. Front. Pharmacol..

[115] Curado N., Dewaele-Le Roi G., Poty S., Lewis J.S., Contel M. (2019). Trastuzumab gold-conjugates: Synthetic approach and in vitro evaluation of anticancer activities in breast cancer cell lines. Chem. Commun..

[116] Li Y., Duo Y., Zhai P., He L., Zhong K., Zhang Y., Huang K., Luo J., Zhang H., Yu X. (2018). Dual targeting delivery of miR-328 by functionalized mesoporous silica nanoparticles for colorectal cancer therapy. Nanomedicine.

[117] Zellmer S., Schmidt-Heck W., Godoy P., Weng H., Meyer C., Lehmann T., Sparna T., Schormann W., Hammad S., Kreutz C. (2010). Transcription Factors ETF, E2F, and SP-1 Are Involved in Cytokine-Independent Proliferation of Murine Hepatocytes. Hepatology.

[118] Tice R.R., Austin C.P., Kavlock R.J., Bucher J.R. (2013). Improving the Human Hazard Characterization of Chemicals: A Tox21 Update. Environ. Health Perspect..

[119] Centre for Drug Evaluation and Research (2017). New Drug Therapy Approvals. https://www.fda.gov/files/about%20fda/published/2017-New-Drug-Therapy-Approvals-Report.pdf.

[120] Yurkiewicz I.R., Muffly L., Liedtke M. (2018). Inotuzumab ozogamicin: A CD22 mAb—Drug conjugate for adult relapsed or refractory B-cell precursor acute lymphoblastic leukemia. Drug Des. Dev. Ther..

[121] Norsworthy K.J., Ko C.-W., Lee J.E., Liu J., John C.S., Przepiorka D., Farrell A.T., Pazdur R. (2018). FDA Approval Summary: Mylotarg for Treatment of Patients with Relapsed or Refractory CD33-Positive Acute Myeloid Leukemia. Oncologist.

[122] Amiri-kordestani L., Blumenthal G.M., Xu Q.C., Zhang L., Tang S.W., Ha L., Weinberg W.C., Chi B., Candau-chacon R., Hughes P. (2014). FDA Approval: Ado-Trastuzumab Emtansine for the Treatment of Patients with HER2-Positive Metastatic Breast Cancer. Clin. Cancer Res..

